# The Mechanism of Metallosis After Total Hip Arthroplasty

**DOI:** 10.1007/s40883-021-00222-1

**Published:** 2021-07-29

**Authors:** Chinedu C. Ude, Caldon J. Esdaille, Kenneth S. Ogueri, Kan Ho-Man, Samuel J. Laurencin, Lakshmi S. Nair, Cato T. Laurencin

**Affiliations:** 1 Connecticut Convergence Institute for Translation in Regenerative Engineering, 263 Farmington Avenue, Farmington, CT 06030-3711, USA; 2 Raymond and Beverly Sackler Center for Biomedical, Biological, Physical and Engineering Sciences, University of Connecticut Health, Farmington, CT, USA; 3 Department of Orthopaedic Surgery, University of Connecticut Health, Farmington, CT, USA; 4 Howard University College of Medicine, Washington, DC, USA; 5 Department of Chemical and Biomolecular Engineering, University of Connecticut, Storrs, CT, USA; 6 Department of Biomedical Engineering, University of Connecticut, Storrs, CT, USA; 7 Department of Materials Science and Engineering, University of Connecticut, Storrs, CT, USA; 8 Institute of Materials Science, University of Connecticut, Storrs, CT, USA; 9 Department of Craniofacial Sciences, School of Dental Medicine, University of Connecticut Health, Farmington, CT, USA; 10 Howard University College of Medicine, Washington, DC, USA

**Keywords:** Metallosis, Mechanism, Biomedical implants, Arthroplasty, Biotribocorrosion

## Abstract

Metallosis is defined as the accumulation and deposition of metallic particles secondary to abnormal wear from prosthetic implants that may be visualized as abnormal macroscopic staining of periprosthetic soft tissues. This phenomenon occurs secondary to the release of metal ions and particles from metal-on-metal hip implants in patients with end-stage osteoarthritis. Ions and particles shed from implants can lead to local inflammation of surrounding tissue and less commonly, very rare systemic manifestations may occur in various organ systems. With the incidence of total hip arthroplasty increasing as well as rates of revisions due to prosthesis failure from previous metal-on-metal implants, metallosis has become an important area of research. Bodily fluids are electrochemically active and react with biomedical implants. Particles, especially cobalt and chromium, are released from implants as they abrade against one another into the surrounding tissues. The body’s normal defense mechanism becomes activated, which can elicit a cascade of events, leading to inflammation of the immediate surrounding tissues and eventually implant failure. In this review, various mechanisms of metallosis are explored. Focus was placed on the atomic and molecular makeup of medical implants, the component/surgical associated factors, cellular responses, wear, tribocorrosion, joint loading, and fluid pressure associated with implantation. Current treatment guidelines for failed implants include revision surgery. An alternative treatment could be chelation therapy, which may drive future studies.

## Lay Summary

Arthroplasty is an invasive procedure which disrupts surrounding joint tissues, and can greatly perturb the joint’s immune homeostasis. In some instances, this may pose a difficult challenge to implant integration. Particles released from implants into the surrounding joint tissues activate the body’s defense mechanism, eliciting a cascade of events, which leads to biotribocorrosion and electrochemical attacks on the implant. This process may lead to the release of even more particles. Besides, implant makeup and designs, frictions between bearing surfaces, corrosion of non-moving parts with modular junctions, surgical mistakes, patient factor, comorbidities, and loosened components can alter the expected function of implants. High accumulations of these ions and particulates result in metallosis, with accompanying adverse complications. Current recommended treatment for failed prosthesis is revision surgeries. However, chelation therapy as a prophylactic intervention may be useful in future efforts but more investigation is required.

## Introduction

Osteoarthritis (OA) is a degenerative and chronic inflammatory disease of the connective tissue that affects cartilage, chondrocytes, extracellular matrix, and the subchondral bone of the joints which most frequently involves the hips and the knees [1]. This may be induced by oxidative stress, inflammatory factors, and mitochondrial dysfunction resulting in DNA damage. When the cartilage within the joint breaks down, the underlying bone begins to change. These changes usually develop slowly with an insidious onset of pain, stiffness, swelling, and reduced joint function [2]. When conservative treatments such as physical therapy and nonsteroidal anti-inflammatory drugs (NSAIDs) to relieve severe osteoarthritis have failed, total joint arthroplasty is considered.

For more than a 100 years, metals and metal alloys have been in use for a host of medical implants [3]. These have also been utilized in joint replacements and have proved to be advantageous over most alternative treatments for end-stage joint osteoarthritis. The most commonly used materials for joint replacement devices include polymers, ceramic composites, and metals [4]. Among these metals are stainless steel (iron-based alloy), cobalt alloys, chromium alloys, nickel, titanium-based alloys (Ti-6Al-4V), molybdenum, and tungsten [5, 6]. The usage of a particular material, metal or alloy, depends on the application. For instance, total hip replacements are subjected to high mechanical loads and must integrate with the host bone; hence, cobalt-based alloys, cobalt-chromium-molybdenum (Co-Cr, Co-Cr-Mo), and cobalt-chromium-tungsten-nickel (Co-Cr-W-Ni) are used widely as a long-term permanent implant due to its high corrosion resistance, higher strength, and hardness [3]. Metal-on-metal (MoM) implants were thought to be advantageous over other procedures due to lower incidence of dislocation; their thin metal acetabular components which could allow for large diameter femoral heads; and an articulation which was anticipated to produce less volumetric wear [7].

In the 1940s, the first MoM hip replacement surgery was performed with a large fixed head made of cobalt-chrome alloy. This gave rise to the first generation of MoM hip implant in 1966 with the McKee-Farrar total hip replacement (THR) prosthesis, and by 1996, more than 1 million MoM articulations have been implanted [3]. At the peak of the procedure, about 10% of all UK hip replacements were MoM, and over half a million patients in the USA have received it [8]. Recent estimate projected that up to 80,000 patients in the UK still have an indwelling MoM prosthesis [9].

Concerns emerged when registries reported increased revision rates of hips replacement procedures. One in eight of all total hip replacements would require a revision within 10 years, and about 60% were due to arthroplasty wear-related complications [9]. Reports showed that metal-related revision for metal-on-plastic (MoP) hips in over 2000 primary THR was 0.5%, while trunionosis had higher rates, with up to a threefold increase in revisions on MoM compared to MoP hips [10]. Smith et al. in 2012 publication called for a ban on all MoM hip implants, based on the data from the National Joint Registry in the UK, which found that MoM hip implants failed at greater rates compared to other types of hip implants [11]. Out of 402,051 hip replacements, 6.2% of MoM hips had failed within 5 years, compared to 1.7% of MoP and 2.3% of ceramics on ceramics (CoC) hip implants [11]. On June 2012, the U.S. FDA issued further regulations on MoM hip procedures, requiring future versions, to undergo clinical trials prior to approval, with subsequent post-market studies to keep them on the market [8]. In the UK in 2014, 83,000 primary hip replacements and 91,000 knee replacements in the National Joint Registry reported revision surgery was required in 8900 hips and 5800 knees [12].

**Fig. 1:**
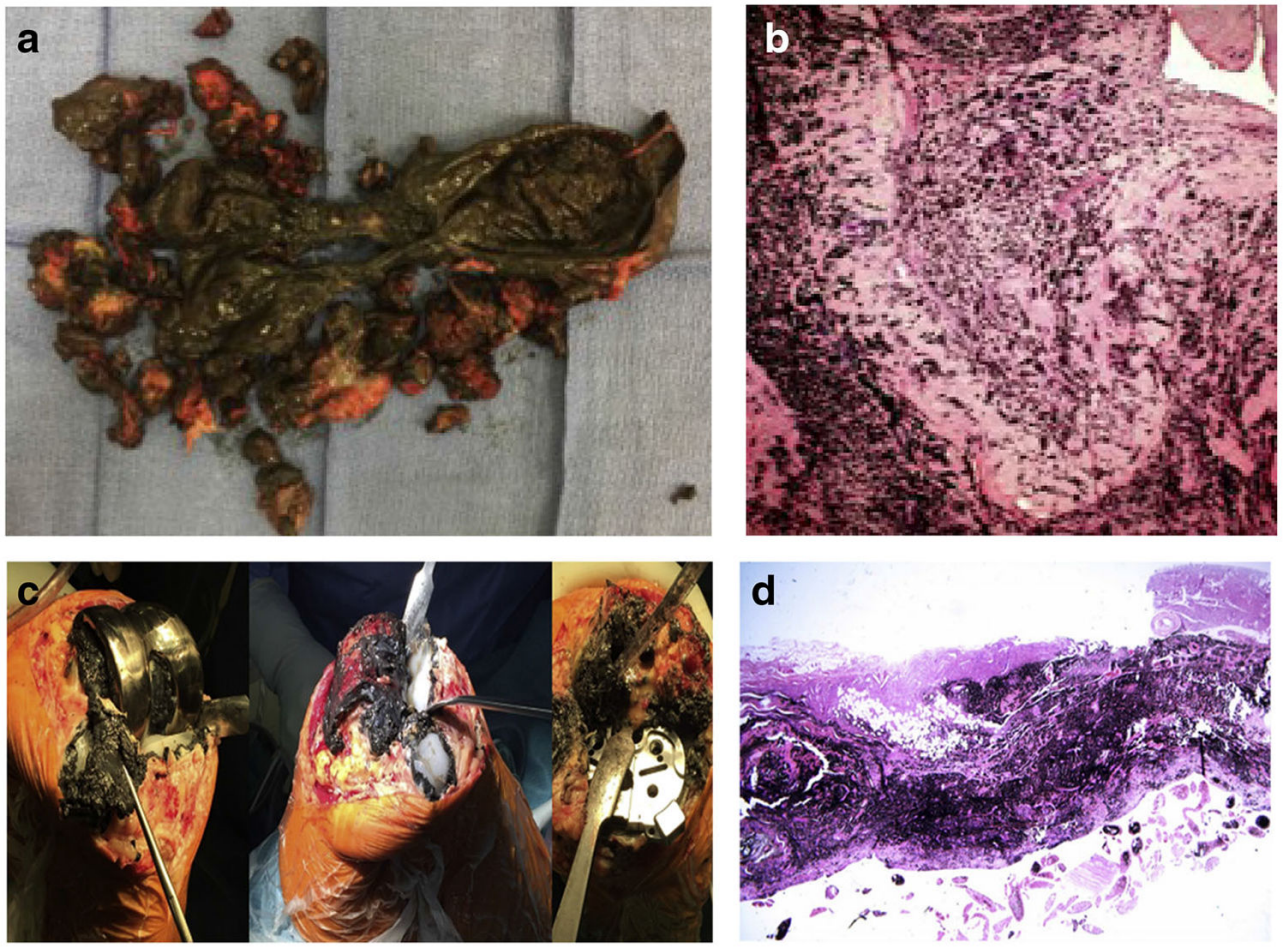
**a** Intraoperative pseudo-tumor in hip arthroplasty. Intraoperative pseudo-tumor showing gross intraoperative findings of extensive pseudo-tumor and dark stained synovium. Pathology reported as fibrovascular tissue and fragments of bone with marked metal wear debris. Thomas et al., 2019, with permission to re-print from Elsevier. **b** Microscopic findings of pseudo-tumor indicating metallosis. Microscopic pathologic findings of pseudo-tumor demonstrating significant metallosis. Thomas et al., 2019, with permission to re-print from Elsevier. **c** Intraoperative photograph of black staining in knee arthroplasty. Intraoperative photographs of the last case showing dark black staining of the synovial tissues and advanced osteolysis with holes filled with metal debris underneath all prosthetic components. Salem et al., 2020, with permission to re-print from Elsevier. **d** Microscopic photograph of metallosis from knee arthroplasty. Microscopic picture showing metallosis-associated synovitis from the wear-related complications. Salem et al., 2020, with permission to re-print from Elsevier

Metallosis results as a syndrome of metal-induced synovitis, which manifests in the abnormal dark macroscopic staining of soft tissues that is associated with abnormal wear from implants (Fig. 1a and c) [2, 13, 14]. The clinical outcomes of revision surgery depend on the amount of bone and tissue necrosis as surgeons replace the MoM implant with a ceramic-on-plastic or plastic-on-metal implant to minimize future complications with metal ions. In addition, a burden is placed on the patient financially. An aseptic revision for THR is estimated to cost £11,897 and £21,937 for a septic case [12]. In the USA, the average cost could range between $50,000 and $100,000, and about €22,759 and €50,000 in most other European countries [15]. By 2010, about 7 million people in the USA have had hip or knee replacement, with a growing prevalence towards the young population [12]. In 2017 alone, it was estimated that about 100,000 THR were performed in the UK, 58,492 in Canada, and 47,972 in Australia, all with an end-stage OA-related complications [15]. More recently, year 2020 projection from the U.S. National Inpatient Sample, together with Census Bureau data, revealed that a total of 498,000 hip and 1,065,000 knee replacements were performed, and this could rise to 1,429,000 hips and 3,416,000 knees by the year 2040 [16].

In MoM hip implants, metal ions and particles are released from the articulation of the metal ball and metal cup during walking or running [15]. Under laboratory conditions, it is estimated that up to 1 mm^3^ of metal particles is worn out from prosthesis per million cycles, generating 10^12^–10^14^ per annum of nanoparticles between 25 and 50 nm [9]. These particles can elicit a local inflammatory response in tissues surrounding the implant by modulating cytokines expression, leading to tissue deterioration (Fig. 1a and c). Local symptoms may manifest as extreme pain which may be associated with bone or tissue necrosis. Pseudo-tumors or a noncancerous buildup of fluid and tissue may also occur around the joint, creating a soft tissue lesion. The local inflammatory response and damage to the surrounding bone and/or tissue has been described as aseptic lymphocyte-dominated vasculitis-associated lesions (ALVAL) [14] (Fig. 1b,and d) [2, 13]. Patients with a progressive ALVAL should be considered for earlier revision to prevent extensive bone, muscle, and nerve damage [17, 18]. Over time, significant instability of implant may develop, which may in turn lead to total failure of the implant.

Less commonly, metal particles can disseminate through the tissues and penetrate cells leading to rare systemic effects [19, 20]. Systemic manifestations include but are not limited to anxiety, depression, mood disorders, skin rashes, neurological complications, cardiomyopathy, visual impairments, and thyroid disorders, leading to weight gain or loss, fatigue, and infections [13]. A detailed list of metals used for implants and their associated benefits and complications at an elevated concentration is shown in Table 1 [3].

There are no specific clinical signs or symptoms that indicate metallosis; thus, a detailed history of symptoms and physical examination are essential for diagnosis. On initial presentation, patients may complain of pain, swelling, skin discoloration, restricted range of motion, and audible crepitus or creaking on weight bearing of the affected joint [30, 27]. The biological exposure threshold for hyper-cobaltemia is levels greater than 1 μg/L, and patients with efficiently MoM implants have average blood cobalt levels of 2 μg/L, and greater than 3 μg/L in those with bilateral hip arthroplasty [20]. Bolognesi et al. recommended 7 parts per billion (ppb) for cobalt or chromium ions as cut-off guide to treatments [7]. However, elevated levels of cobalt ions can be detected in patients experiencing no symptoms [17]. In addition to these, computed tomography (CT) scans and magnetic resonance imaging (MRI) can help determine if local tissue is impacted and assess potential ALVAL. With regard to THA, plain radiographs demonstrating a curved radiolucency under the femoral neck, broken tines, or a worn-through liner may be indicative of metallosis. In more severe cases, disposition of metal wear may produce opacification of the periprosthetic soft tissue which can produce a radiographic bubble sign, and diagnosis is further confirmed by aspiration of rusty fluid from the affected joint [31].

The mechanisms of metallosis have been debated among researchers within the orthopedic community since its onset. These efforts have been focused on developing an understanding of the unusual adverse outcomes of arthroplasty in order to guide specific therapy. With the increasing demand for total hip arthroplasty and the rising rates of revision, it is essential to properly define and describe the mechanisms that contribute to these failures. This manuscript highlights recent reports on the mechanism of metallosis with a focus on atomic and molecular makeup of medical implants, the component/surgical associated factors, wear and cellular responses as well as the U.S. FDA’s publication on Biological Response to Metal Implants and recommendations.

## Factors Contributing to Metallosis

### Atomic and Molecular Makeup of Medical Implants

Metallic biomaterials are suitable for orthopedic implants due to their mechanical properties allowing them to withstand heavy loads. However, inside the body, biological fluid containing hydrogen molecules, oxygen molecules, proteins, and ions is extremely corrosive on metals, causing undesired reactions with the surrounding tissues [18]. Implanted material can be categorized as corroding, passive, or immune to the body depending on their molecular structure. Most orthopedic implants, including cobalt-chromium alloy, fall under the passive category [21]. These metals usually form a protective oxide layer that inhibits attack by the body’s fluids. To improve their osteointegration qualities, surface treatments such as machining, acid etching, electro-polishing, anodic oxidation, sandblasting, and plasma spraying are customarily incorporated into the manufacturing processes of orthopedic implants [3]. This serves as a method of protection, attenuating and diminishing the reaction at the interface of the implanted material and the surrounding physiological environments. However, despite these measures, other factors contribute to the inflammatory response.

The effectiveness of an oxide layer as a protective barrier is a function of the oxide chemistry, thickness, and integrity. An oxide layer should be approximately 10 nm thick, chemically stable, uniform, defect-free, and adhere well to the metal substrate, with no subsurface intermetallic layer [22, 23]. A uniform and defect-free oxide layer would provide optimal protection, since the release of metal ions and body fluid’s reaction can occur across the entire surface area of a device; and implant oxide layer modifications to remove subsurface intermetallic layers as well as gross manufacturing surface defects have been efficacious [3, 22].

Cobalt (Co) alloys possess properties of high-temperature, good wear, and fatigue resistance. They generally have superior resistance to mechanical fatigue by the formation of a chromium oxide (Cr_2_O_3_) film that acts as a passive film in response to electrochemical reactions [21]. Pure cobalt has a corrosion behavior that is similar to nickel with a slight advantage of lower susceptibility to body fluids; cobalt-chromium-molybdenum (Co-Cr-Mo) alloys have good compatibility and are tolerated by the body, while a combination with nickel shows a higher coefficient friction both in bearing surfaces [28]. Typically, the thin fibrous Cr_2_O_3_ film layer is formed at the interphase when these Co alloys are implanted in direct opposite to bone; however, this film layer is liable to damage and degradation with motion and activities [28].

**Fig. 2:**
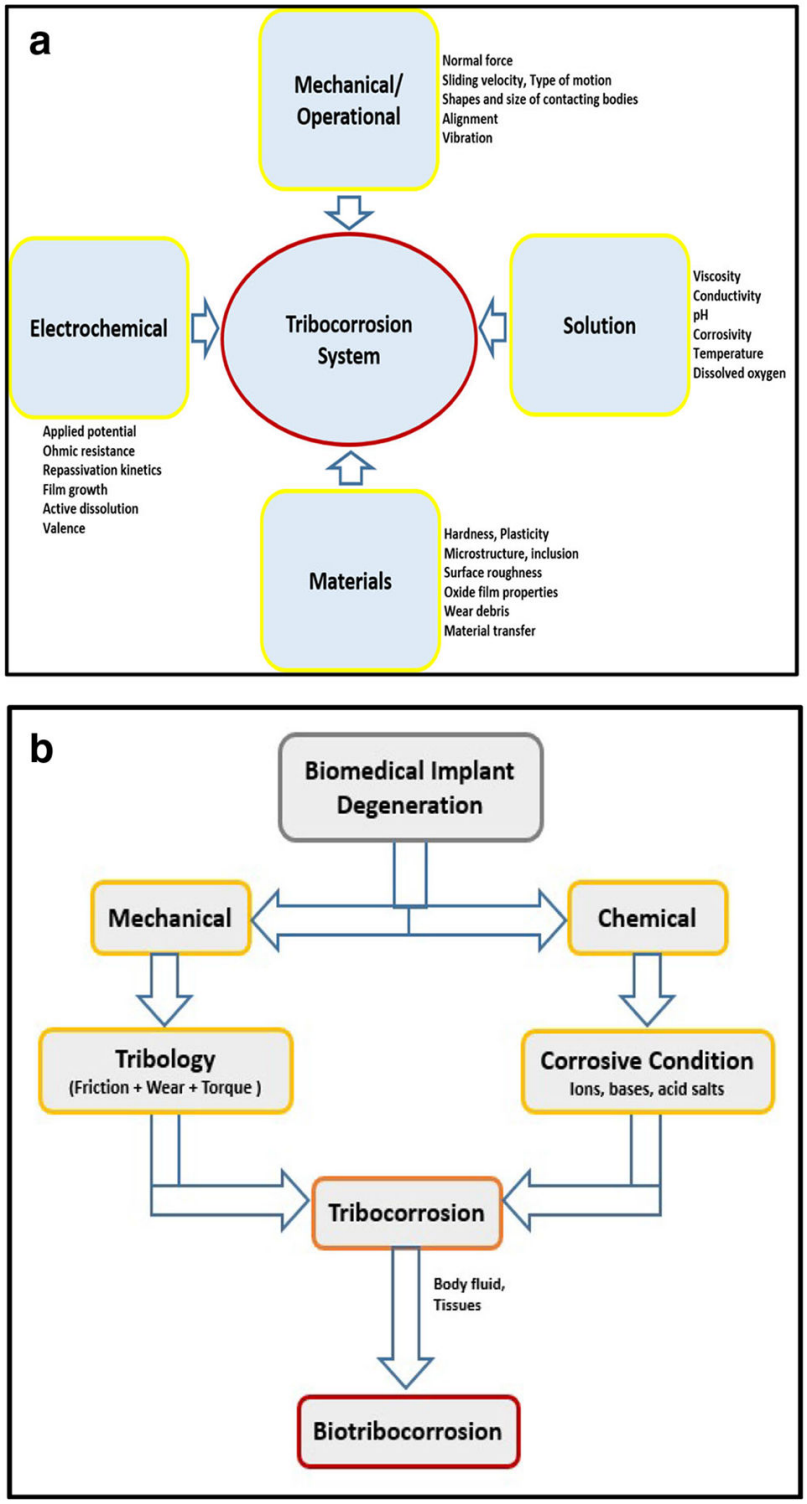
**a** Factors influencing tribocorrosion. The schematic diagramof the tribocorrosion system depicting the various components that act in synergy to cause the irreversible transformation of materials, functions, and status as a result of concurrent mechanical, chemical and electrochemical interactions between surfaces in relative motion. **b** Concept of biotribocorrosion. The schematic diagram of the biotribocorrosion phenomena that deals with mechanical loading and electrochemical reactions occurring between elements of the tribological system when exposed to biological environments, like body fluid

Figure 2a illustrates local physical factors that play a role in the survival of a medical implant, such as pH, ion concentration, molecules, etc. Overall, cellular activities and proteins at the implant site first adsorb onto the metal surfaces aiding cell-metal interaction, and then, macrophages, monocytes, neutrophils, and other immune cells can secrete reactive oxygen species and cause a drop in local pH, which increases susceptibility to body fluid [22].

### Surgical Parameters, Implant Interface, and Implant Design

The amount and type of wear debris ensuing from an arthroplastic joint depends partly on the combination of weight-bearing materials making up the gliding surfaces of the prostheses [24]. Prostheses can be made up of a metal femoral head (cobalt-chromium, ceramics) on ultrahigh molecular weight polyethylene (UHMWPE) and a metal head on a metal acetabular cup which is usually a Co-Cr-Mo alloy; this combination is commonly referred to as a metal-on-metal (MoM) implant [28]. Another popular configuration is a ceramic (alumina and zirconia based) head on a ceramic acetabular cup, which known as ceramic-on-ceramic [28, 32]. MoM articulating implants have been identified as a major cause of metallosis and ALTR. However, in addition to the materials that comprise the prosthesis, there are a few additional characteristics of the implant which contribute to the mechanism of metallosis.

#### Modular Design

Modular implants are widely used due to the flexibility that it provides a surgeon intraoperatively as it can be easily adjusted based on the patient’s anatomy; however, multiple modular connections within a single hip system can predispose to wear, as the modular interface at the head-neck junction requires an elevated measure of trunionosis to improve self-interlocking between components [12, 25]. Surface roughness increases contact stress and scratches from the cyclic relative motion of patients during activities; the cyclic motion inflicts mechanical damages to the surface of the head-tapper junction/neck-body junction and accelerates the rate of particle release within surrounding tissues [12]. Metal ions can be generated not only at the junction between the femoral head and socket, but also at the head and stem of femoral components, and the junction between modular components in MoP [33]. Contact surfaces can also create crevices at the modular implant interface which can lead to corrosion. The small volumes of modular space limits sustained aeration of the joint fluid and the continuous oxidation leads to a decrease in the local pH level by the consumption of dissolved oxygen in the synovial fluids [17].

#### Polymer Cement Interface

In total hip and knee arthroplasty with a polymer cement interface between the implant and the bone, the cement mantle is expected be stable, intact, and firm so that it ensures a well-adaptive physiological bone-remodeling response [34]. However, sometimes, fibrosis may occur between the cement and the implant; and the extent of this fibrosis between implant surface and the cement mantle may enable de-bonding or fatigue fracture spaces which contribute to metallosis from gradual loosening, instability, and wear particles release [35]. Routinely, implants contain a porous coating for protection against fatigue failure that can occur following the degradation of the cement layer around a total hip arthroplasty. This procedure increases both fixations with the cement and promotes osteointegration to the surrounding cement-less prostheses; however, biocompatibility reports relating to this manipulation cause an apparent higher release of metal ions to the surrounding tissues [4].

In non-cemented implants, there is a special design feature and coating technology that enables bone interdigitating in-growth to the porous surface of implants; the coating can modulate bone in-growth depending on the rate of remodeling of the original existing bone [34]. The shape of the implant and the tight micromechanical locking, commonly known as the “press-fit” of an implant to the bone bed, are paramount to success. Bone in-growth and on-growth may be enhanced by the use of bioactive coatings such as calcium phosphatase and hydroxyapatite. However, excessive coating can fail under fatigue, leading to the unrestricted release of wear particle and metallosis [34].

#### Implant Articulation Design

In total hip replacement procedures, the use of mega-head total hip replacement implants with taper junctions has raised metallosis-related concerns [11, 12, 16]. Ideally, metal-on-metal (MoM) implants diminish the linear and volumetric wear of the bearing surfaces that exist on metal-on-plastic (MoP) hip implants; these two procedures, however, have common grounds in the head/cup size, the orientation of the cup and neck, and the mechanism of lubrication regardless of implant articulation type [11, 36]. Large-head MoM total hip arthroplasty was introduced to provide stability and increase the potential for low wear [18]. However, it was later revealed that a mega head in total hip replacement results in increased friction within the bearing causing an increase in frictional torque [4]. Increased frictional torque stresses the taper junction where the generated force and electrochemical actions cause increased release of metallic particles and ions into the tissues surrounding the implants. This also affects the fixation of bone causing instability and failure. Large-head total hip replacements have had more metallosis-related complications and failure rates than hip resurfacing devices of the same size [3]. In contrast to the result of a large head on total hip arthroplasty (THA), MoM hip resurfacing with a smaller femoral head size has increased risk of mechanical deterioration and edge loading with high release of metal particles. Mobile-bearing knees has also been associated with an increased rate of metallosis due to increased release of smaller particles and granular debris compared to fixed bearing knees [6, 19].

#### Surgical Procedural Mistakes and Errors

Surgical mistakes, inexperience, and procedural challenges have been implicated in metallosis [3]. Revision surgeries replacing a failed plastic or ceramic implant with metal-on-metal total hip arthroplasty have posed a high chance of 3rd body wear [2]. A truncated acetabular component has also been linked to metallosis, owing to accelerated mechanical wear from 3rd body, which usually occur at an articulation between intentional bearing surfaces in the presence of wear debris. These may include the femoral head and the acetabular cup in the presence of PMMA cement debris, metallic debris, or hydroxyapatite particle [34]. The differences in the linear wear rates post-surgery are quite significant depending on the biomechanical loading. Lateralization of the femoral component can help to restore the preoperative hip biomechanics and significantly decrease wear. In total knee arthroplasty, it is also speculated that malrotation of the femoral component contributes to the formation of wear particles and diffusion of knee pain, knee joint laxity in flexion, and patellar maltracking [37, 38].

A suboptimal cup inclination angle greater than 50–55° also contributes to metallosis [6]. In hip replacement surgeries, a low femoral offset and an acetabular angle greater than 45° can enhance wear particle formation from the acetabular [12]. This increase wear associated with failure of lubrication within the prosthesis was linked to edge loading. Edge loading locally increases the contact pressure between the implant surfaces leading to the failure of the pressure film lubrication, thus increasing mechanical wear causing the formation of debris [3].

### Biotribocorrosion-Associated Factors

Tribocorrosion is the concept of material degradation due to a combined effect of corrosion and tribology principles, especially wear. While tribology is concerned with friction, lubrication, and wear, corrosion deals with the chemical and electrochemical interactions between materials and their environments [24, 39] (Fig. 2a). Corrosion and wear may act synergistically. Narrowing this concept in vivo gives biotribocorrosion. Biotribocorrosion deals with mechanical loading and electrochemical reactions occurring between elements of the tribological system when exposed to biological environments [21]. The application of biotribocorrosion affects most medical implants by leading to the release of wear particles and metallosis, which have been identified as the chief factor of joint replacement complications (Fig. 2b).

As mentioned earlier, the reactivity category (chemical and electrochemical potentials) of most metals and alloys used in the manufacturing of biomedical implants is usually either passive or immune to body fluid [3]. The vast majority used for medical implants falls within the passive category. Tribocorrosion is most critical for these corrosion-resistant passive metals [40]. These metals are thermodynamically unstable in the presence of oxygen or water and derive their corrosion resistance capacity from a thin oxide film present on their surface called passive film [21]. Passive film acts as a protective barrier between metals and their electrochemically active environments, and the thickness of these passive films is usually a few atomic layers; however, these microscopic sheets can provide the desired protection to a biomedical implant in its electrochemically hostile in vivo environment [21]. In the event of damage/compromised integrity, the passive films can self-heal spontaneously by metal oxidation. However, when the implant surface is subjected to severe rubbing through streams of impacting particles, the damage becomes continuous and extensive causing the self-healing process to not function. Subsequently, a higher rate of metal oxidation would be required to restore the proof status [41]. With this, the underlying medical implant will experience strong electrochemical attacks as a result of synergy between mechanical and chemical reactions. The damages will lead to an extensive restoration time for the protective passive film if it can be restored [3].

### Postoperative Joint Loading and Fluid Pressure

Synovial fluid lubricates native joints, provides nutrients to surrounding tissues, and protects the joint from large compressive forces exerted by the weight of the body [16]. Artificial joints operate in the synovial solution that forms a fluid film lubrication at the joint interface. When there is sufficient pressure of the synovial fluid and a conforming design, the joint bearing helps to establish stable hydrodynamic lubrication during active motion [24]. However, in the case of insufficient pressure, the joint fluid may develop an unstable lubrication film causing kinetic friction increase and gradual damage to the joint surfaces. This mechanical reaction usually acts in synergy with other electrochemical and biological reactions to increase the release of a metal particle and implant deterioration [24].

Fluid flow, shear forces, and hydrostatic pressures most often regulate the function of the cellular and physiological activities within the joint space and contribute to pathology of periprosthetic bone. The pressure mechanisms are associated with biomechanical loading, which is dependent on the lifestyle and physical activities of the patient as well as the type, orientation, and position of the implant. Usage, as well as duration, is a known factor of failure; both overusage and underuse can pose a serious problem [17, 18]. During walking, running, squatting, and other physical activities requiring the joints, fluid pressure waves are generated due to cyclic pressure variation and cyclic volume in the hip and knee. The physiological movement in an arthroplastic hip or knee joint causes interscapular pressure alternations. Cyclic loading during gait functions as a pump that distributes joint fluid containing particles through the port of least resistance [37].

The initial joint space between the head and the cup may vary in size depending on the patient and predisposed physical activities [34]. When the joint volume is decreased, fluid pressure increases and begins to tear the interface between the implant and host, which affects the outcome of joint replacement surgery. Edge loading locally increases the contact pressure between implant surfaces leading to failure of pressure film lubrication and consequently increases wear [12]. These actions impact greater loading and multi-scale displacement between the modular joint components [4].

**Fig. 3:**
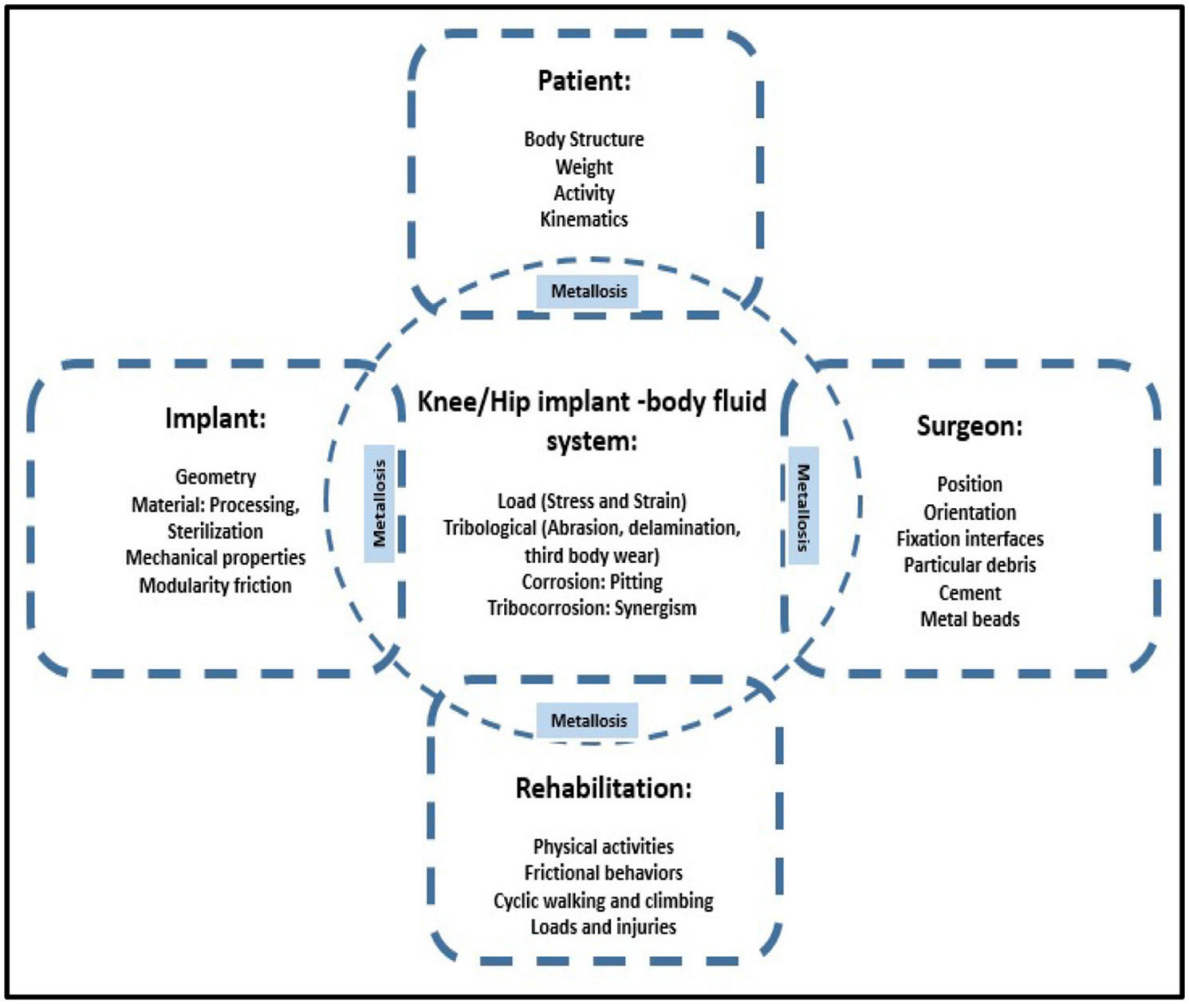
The contributing factors to metallosis. A schematic diagram showing the various contributing factors that act alone or in a combined effect on implants and body fluid systems to induce metallosis

Overall, the rehabilitation process of implant surgery as well as the patient’s makeup and conditions can contribute to particle release and metallosis (Fig. 3). Genetics, lifestyles, allergies, preexisting ailments, acquired metal sensitivity and allergy, female gender, individuals with bilateral hip/knee implants, obesity, underweight, renal insufficiencies/failures, and highly physical jobs that require heavy lifting have all been implicated in the mechanism of metallosis [9, 12, 26, 29].

### Cellular Responses to Metallosis

Medical implants are certified as biocompatible but poor bone/implant contact predisposes the surfaces to inflammation and fibrosis [3]. The response of the body and fate of implants vary widely according to the patient, site of implantation, type of biomaterial, components, and the degree of trauma created during implantation [34].

After an arthroplasty, proteins and other biomolecules present in the blood and biological fluids rapidly adsorb onto the surface of the implant causing changes in vascular flow, caliber, and permeability [42]. In most instances, adsorbed fibrinogens, immunoglobulin G, and complement fragments mediate leukocyte-biomaterial interactions and inflammatory reactions; acute inflammation occurs similar to foreign body reaction (FBR), which is characterized by the secretion and infiltration of numerous inflammatory cells and chemical mediators [43]. The inflammatory phase gives rise to the granulation of tissues that end up in the formation of the fibrous capsules comprising of the collagen matrix, fibroblasts, and fibrocytes [28]. During the acute phase, polymorphonuclear leucocytes infiltrate the implant sites followed by monocytes and macrophages. The layer of surface-adsorbed proteins modulates macrophage phenotypes and subsequent functions of phagocytosis and cytokine expression. Vascular endothelial cells and fibroblasts in the implant site proliferate leading to the formation of granulation tissue [26, 44]. The end stage of these reactions is the formation of a fibrous capsule around the implant with little restitution of normal tissue structure. Depending on patients’ inflammatory stimuli, chronic inflammation and insufficient healing of local tissue at the device interface can occur. The presence of mononuclear cells, monocytes, plasma cells, and excessive collagen deposition around the implants leads to ALTR and eventually metallosis [12, 45]. The peak of such chronic responses is the fusion of monocyte-derived macrophages to form multinucleated foreign body giant cells (FBGCs); thus, this complex process involves a myriad of molecules and ions’ reactions, creating a fibrous encapsulation and a physiochemical barrier that severely limits device integration [13, 43].

### Macrophages and Pattern Recognition Cascade

Wear particles are coated with endogenous carrier proteins, which may alter their identity and are recognized by the immune system as foreign body haptens [3, 34]. However, wear particles alone can be recognized as antigens in certain instances [43]. The uptake of particulate metal debris by macrophages through phagocytosis is a key process by which implants may trigger inflammatory responses leading to metallosis [28, 29, 44]. After macrophages identify particles through pattern recognition receptors, including toll-like receptor, retinoid acid-inducible gene-1-like receptors, aim 2-like receptor, and nucleotide-binding oligomerization domain-like receptors, they form a cascade of reactions generating pro-inflammatory cytokines, including interleukin-1β, interleukin-6, interleukin-18, and tissue necrosis factor-α, as well as the chemokines (CXCL8, CCL2, and CCL3), and other small molecule inflammatory mediators [31, 42].

At the implant site, macrophages are activated both by implant debris and damage-associated molecular patterns (DAMPs) released upon tissue injury and cell death signaling through cryopyrins such as NLRP3, inflammasomes, and (toll-like receptor) TLR pathways [12, 45]. Activation of the NLRP3 complex is dependent upon the size, shape, and chemistry of debris. Phagocytized particulate less than 10μm is endocytosed and transported to lysosomes where the acidic microenvironment of these vesicles promotes corrosion and stimulation of further release of metal ions. Metal ions and particulates have been shown to directly activate toll-like receptor-4 (TLR4) promoting local inflammation and tissue remodeling through NF-kβ-mediated cytokine production [45].

Altogether, these mechanisms display that early innate sensing of implant debris can be performed by multiple pathways, both direct through receptor recognition of metal ions, particulates, and surfaces and indirect through recognition of alarmins released by tissue injury [3].

### Lymphocytes and Cellular Complexes

Infections and sepsis can occur following arthroplasty, which can predispose implants to metallosis by triggering abnormal osteoclast bone resorption, causing malalignment/instability of implant [45-47]. Surgical site infections can be superficial or deep to the implant, and tissues surrounding an infected implant customarily contain monocytes, macrophages, neutrophils, and B lymphocyte and T lymphocyte infiltrates [31].

Indeed, the peri-implant tissue of septic loosening is typically infiltrated by various lymphocyte subpopulations, macrophages, fibroblasts, and osteoclasts; and this has led to added investigation of lymphocyte subpopulation, especially B and plasma cells, as useful diagnostic markers of implant-related infection [46]. B lymphocytes from patients with metal implants have also been reported to be more active and stimulated [3]. The role for B cell-mediated immunity in response to metal could be through its antibody production against metal haptens mediating types I, II, and III hypersensitivity reactions [48, 49]. Histological reports have indicated signs of B cell activation in tissues associated with failing metal implants, especially the co-stimulatory molecules of B cell-activating factor TNFSF13B and a proliferation-inducing ligand TNFSF13 [3, 34].

Bacterial endotoxins and self-molecules released as a result of cellular activation and tissue necrosis can adhere to implants, which can accelerate inflammatory host response and wear [47]. Lipopolysaccharide, the main antigenic component of gram-negative bacterial outer cell membranes, adheres to polyethylene and metallic particles inducing accelerated activation and inflammatory responses [12]. According to commonly accepted paradigm of aseptic loosening, wear particle-induced and macrophage-mediated inflammatory response cause peri-implant osteolysis. Wear particle-activated macrophages secrete chemokines and pro-inflammatory cytokines that lead to further macrophages recruitment, increase osteoclastogenesis, and suppression of osteoblast, thus creating microenvironment that favors bone resorption over formation [45, 48]. Bacteria-derived pathogen-associated molecular patterns (PAMPs) and damage-associated molecular patterns (DAMPS) can activate toll-like receptors (TLR) and their receptors, similar to metal ions including cobalt and other wear particles [19, 28, 46, 49]. Attachment of MAMPs or PAMPs to particles as haptens can activate macrophages against wear debris [48]. Exogenous MAMPs-like ligands binding to the surfaces of the wear particles can be recognized by pathogen recognition receptors (PRRs) and activate PRR-equipped cells leading to very potent stimulation of osteoclasts absorbing the bone surrounding the infected implant [45].

In vitro studies have also shown that human osteoclasts can corrode stainless steel and titanium leading to the production of metal ions responsible for inflammatory reactions [47]. Traces of cellular activities on metallic explants have been reported as inflammatory cell-induced (ICI) corrosion that occurred as the result of cells sealing on the metal surfaces and releasing reactive oxygen species (ROS) through Fenton-like reactions [47]. In a cohort study of 100 retrieved Co-Cr hips implants, Di Laura et al. demonstrated the clinical evidence of corrosion, consistent with these cellular mechanisms. It was found that 59% of explants had evidence of surface damages consistent with ICI, which had a significant association between the patterns and aseptic loosening for ASR modular and the Durom modular [47].

### Mast Cells Response Leading to Extracellular Fibrosis

Mast cells regulate homeostasis associated with the development of fibrosis as they initiate fibroblast activation and extracellular fibrosis. In allergic reactions, they cause immunoglobulin E (IgE) receptor aggregation leading to exocytosis of degranulation mediators [43]. Metallic implants can initiate hypersensitivity with marked increase in macrophages oxidative responses [48]. The accumulation of arthroplastic wear particles in the joint recruits mast cells, which subsequently release serine protease, tryptase, and cytokine IL-4 that mediate implant debris-induced fibrosis [43].

There have been some other reports on the contribution of mast cell in bone-implant tissue interface biology, as influx of both activated and degranulated mast cells are observations found in many of the retrieved implants [43, 50-52]. Both activated and degranulated mast cells encourage fibrotic collagen deposition; thus, it was hypothesized that the particulate debris could provide stimulus for mast cell activation, which further leads to release of interleukins including IL-4 and tryptase [43].

This cascade of mast cell activation and contribution from other inflammatory cells could have a profibrotic response resulting in the fibrotic collagen capsule formation. Thus, the collagen capsule also has the ability to induce collagenase, other extracellular matrix enzymes, and biochemical factors that could lead to bone degradation, aseptic loosening, and metallosis from malalignment of implants [43, 45].

### NF-kB Activation Leading to Inflammatory Osteolysis

NF-kB signaling can be activated in macrophages and osteoclasts by exposure to wear particles, leading to the hypothesis that targeting NF-kB activity could be a potential strategy to mitigate metallosis preceding osteolysis [45]. Wear particles can trigger this process by stimulation of osteoclast precursor NF-kB and MAP kinase pathways. These activate the upstream transforming growth factor beta-activated kinase-1, a key regulator of signal transduction cascades that activate NF-kB and AP-1 factors [4].

**Fig. 4:**
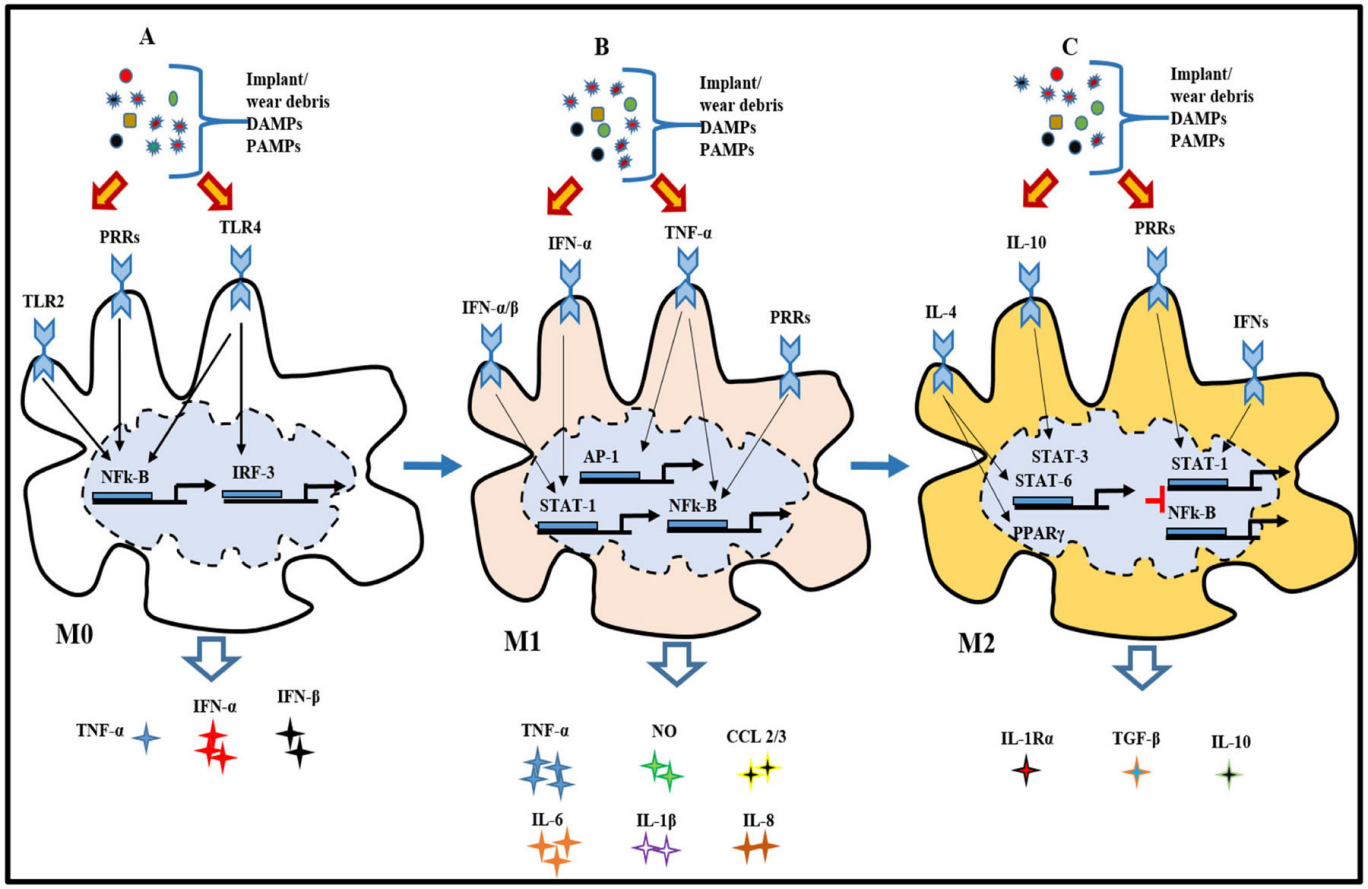
NF-kB activation effect. The schematic diagram showing the response of NF-kB activation by wear debris. NF-kB signaling can be activated in macrophages and osteoclasts by exposure to wear particles. Under the influence of Th1 and Th2 cell-derived cytokines, primary macrophages (M0) assume two distinct phenotypes known as classically activated macrophages (M1) and alternatively activated macrophages (M2). M1 activation can be induced by IFN-g secreted by NK or Th1 cells, TNF-a receptors, implant/wear debris, DAMPs, and PAMPs. Working in synergy, IFN-g and TNF-a signal Janus kinase transducer and activator of transcription (JAK-STAT) pathway, thus activating the transcription factors STAT1 and IRF5 that primarily transcribe M1-related genes. Acting as autocrine/paracrine effect, these can partially substitute for IFN-g in inducing and sensitizing the M1 cells. M1 activation is further characterized by production of high levels of IL-12 that supports pro-inflammatory cytokines (TNF-a, IL-1b, IL-6, and IL-23) and inflammatory chemokines (CCL2, CCL3, CCL4, IL-8, CXCL9, CXCL10, and CXCL11) that recruit neutrophils, monocytes, and activated Th1 lymphocytes. Furthermore, macrophages can assume the M2 cells. M2 activation occurs when M0 or M1 is exposed to Th2 cytokine, such as interleukin-4 (IL-4). Thus, M2 activation is characterized by the suppression of pro-inflammatory cytokine production, antigen presentation ability, and the production of increased levels of IL-10 instead of IL-12.

Macrophages are a dynamic and adaptive population of cells that can assume various phenotypes following instructions by signals derived from the local microenvironments, as most tissues contain highly specialized macrophages subpopulations [49, 51]. These resident microphages (M0) are responsible for the quiescent removal of apoptotic cells, regulation of homeostasis, and various tissue-specific functions. Under the influence of Th1 and Th2 cell-derived cytokines, M0 assume two different phenotypes, known as classically activated (M1) macrophages and alternatively (M2) activated macrophages [45]. M1 activation can be induced by two pro-inflammatory signals: (1) interferon-g (IFN-g) secreted by NK or Th1 cells and (2) tumor necrosis factor-alpha (TNF-a) receptors [48]. Working in synergy, IFN-g and TNF-a signal Janus kinase transducer and activator of transcription (JAK-STAT) pathway; thus activating the transcription factors STAT1 and IRF5 that primarily transcribe M1-related genes [45, 48]. Other factors that activate M1 cells include implant/wear debris, damage-associated molecular patterns (DAMPs), and PAMPs [32, 43, 51]. These are recognized by TLRs, leading to NF-kB activation, production of type 1 interferon, as well as various pro-inflammatory factors. Acting as autocrine and/or paracrine effect, these can partially substitute for IFN-g in inducing and sensitizing the M1 cells to pathogen recognition by upregulation of TLRs, inflammasomes, Fc receptors, etc. [45, 48, 49]. Major histocompatibility complex (MHC) and co-stimulatory molecules are also upregulated by antigen presentation and processing [28]. M1 activation is further characterized by production of high levels of IL-12 that supports pro-inflammatory cytokines (TNF-a, IL-1b, IL-6, and IL-23) and inflammatory chemokines (CCL2, CCL3, CCL4, IL-8, CXCL9, CXCL10, and CXCL11) that recruit neutrophils, monocytes, and activated Th1 lymphocytes [12, 43, 45]. Furthermore, macrophages can assume the M2 cells, a heterogeneous subpopulation that participate in a wide range of physiological and pathological processes such as Th2-polarized responses, tissue healing, homoeostasis, fibrosis, etc. M2 activation occurs when M0 or M1 is exposed to Th2 cytokine, such as interleukin-4 (IL-4) [45, 48, 49]. IL-4, which is produced by eosinophils, mast cells, basophils, and activated Th2 lymphocytes, binds to an IL-4 receptor that signals through the JAK-STAT6 to activate the peroxisome proliferator-activated receptor-gamma (PPARg) and phosphoinositide 3-kinase [45]. PPARg thus exerts a direct suppressive effect on the production of inflammatory cytokines mediated by STAT1, activator protein-1 (AP-1), and NF-Kb [45]. Thus, M2 activation is characterized by the suppression of pro-inflammatory cytokine production, antigen presentation ability, and the production of increased levels of IL-10 instead of IL-12 [14, 48, 49] (Fig. 4) [45]. To be concise, when this cascade of events is activated, macrophages and other cells in the implant bed upregulate NF-kB, a transcription factor with strong control over pro-inflammatory factor synthesis and osteoclastogenesis response [48, 51].

## Discussion

**Fig. 5:**
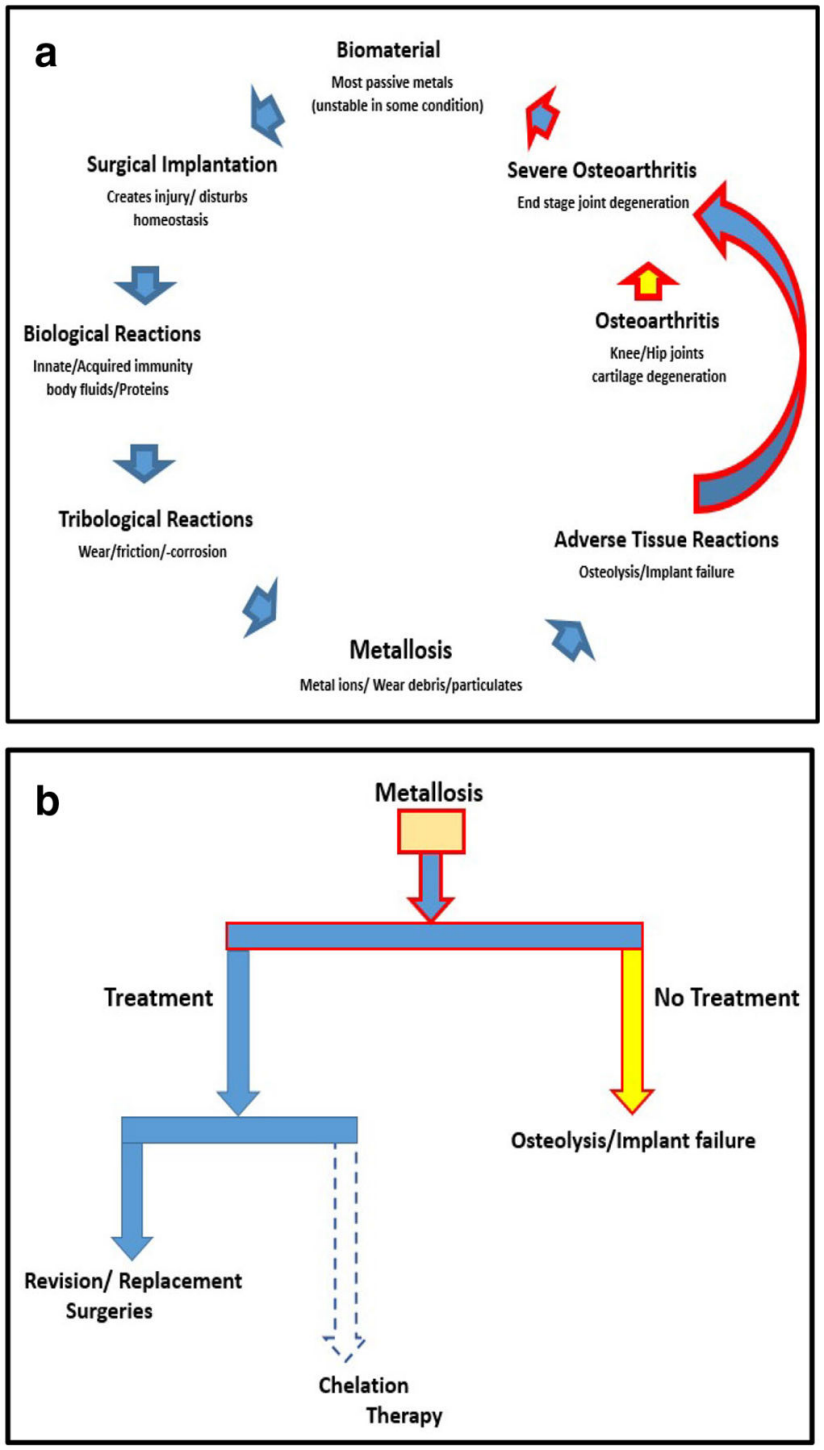
**a** The summary of metallosis originating from arthroplasty. A schematic diagram showing the cycle of events and conditions leading to arthroplasty, metallosis, osteolysis, and revision surgery. **b** Treatments of arthroplasty-related metallosis. A schematic diagram showing the current treatments of metallosis and the result of no treatment

Joint replacements have been among the most successful surgical procedures for end-stage osteoarthritis, relieving severe pain and improving joint function, thus returning patients to normal lives; nevertheless, reports estimated that about one in eight (12.5%) of all total hip implants would require revision within 10 years and 60% of these owing to wear-related complications [9] (Fig. 5a). Metallosis after total hip arthroplasty is a subject that has attracted high research in recent years after the reported association with potential adverse outcomes [2]. As of March 2019, there are no FDA-approved metal-on-metal total hip replacement devices currently on the market in the USA. However, there are two FDA-approved MoM hip resurfacing devices available with the recommendation that young males with large femoral heads are the best candidates for MoM hip resurfacing systems [3].

In normal functioning joint, blood concentration of cobalt and chromium ions rises after insertion of MoM prosthesis, which usually reach to maximum within first year post-operation and then wane in subsequent years [9]. Elevation of metal ions in the blood is an important aspect of the diagnosis; however, high metallic ion blood concentration does not always correlate with established metallosis and symptoms [20, 26].

In tracing the mechanism of occurrence, surgical implantation alone injures tissues and greatly perturbs the smooth homeostasis of joints [12, 17, 53]. Tissues’ immune response during wound healing at postoperative periods affects the activities of implants and the surrounding tissues [17, 45]. Although most biomedical implants have been deemed safe, sterile, and biocompatible by FDA [3], various biochemical and physiological systems become activated under specific conditions [13, 44]. However, the body’s biological effect on implant material is by some means dependent on the biodegradable mechanism by which it releases particles into the surrounding tissues as well as patient factors. Subsequently, these inflammatory responses elicited are the result of a chain of events initiated as the natural body defense mechanisms to an intruding medical implant, thus leading to heightened corrosion rate of components and wear [13, 46]. Accumulations of these particulates are associated with accompanying adverse reactions and complications, leading to prosthetic loosening, osteolysis, and complete failure of the implant [13, 19, 26, 42, 47, 51]. The current treatment guidelines to avoid further shredding of the implant particles into tissues are revision surgery (Fig. 5b). After removal and replacement, the body’s buildup of metal ions and particles will drop gradually; however, patients may develop hypersensitivity to a low level of metals [6, 12].

## Future Directions

Recent developments in materials chemistry, metallurgy, and manufacturing continue to drive innovation in the design and diversification of more compatible components. Even though great improvements in the combination of materials have been achieved, more work is needed. Current knowledge of biomedical implant technologies has not yet reached the expected goals. Therefore, it is imperative for continuous research of implants with better designs to speed up osteointegration with biologically based coating for effective wear, fatigue resistance, and low host-immunoreactivity [6, 21].

**Fig. 6:**
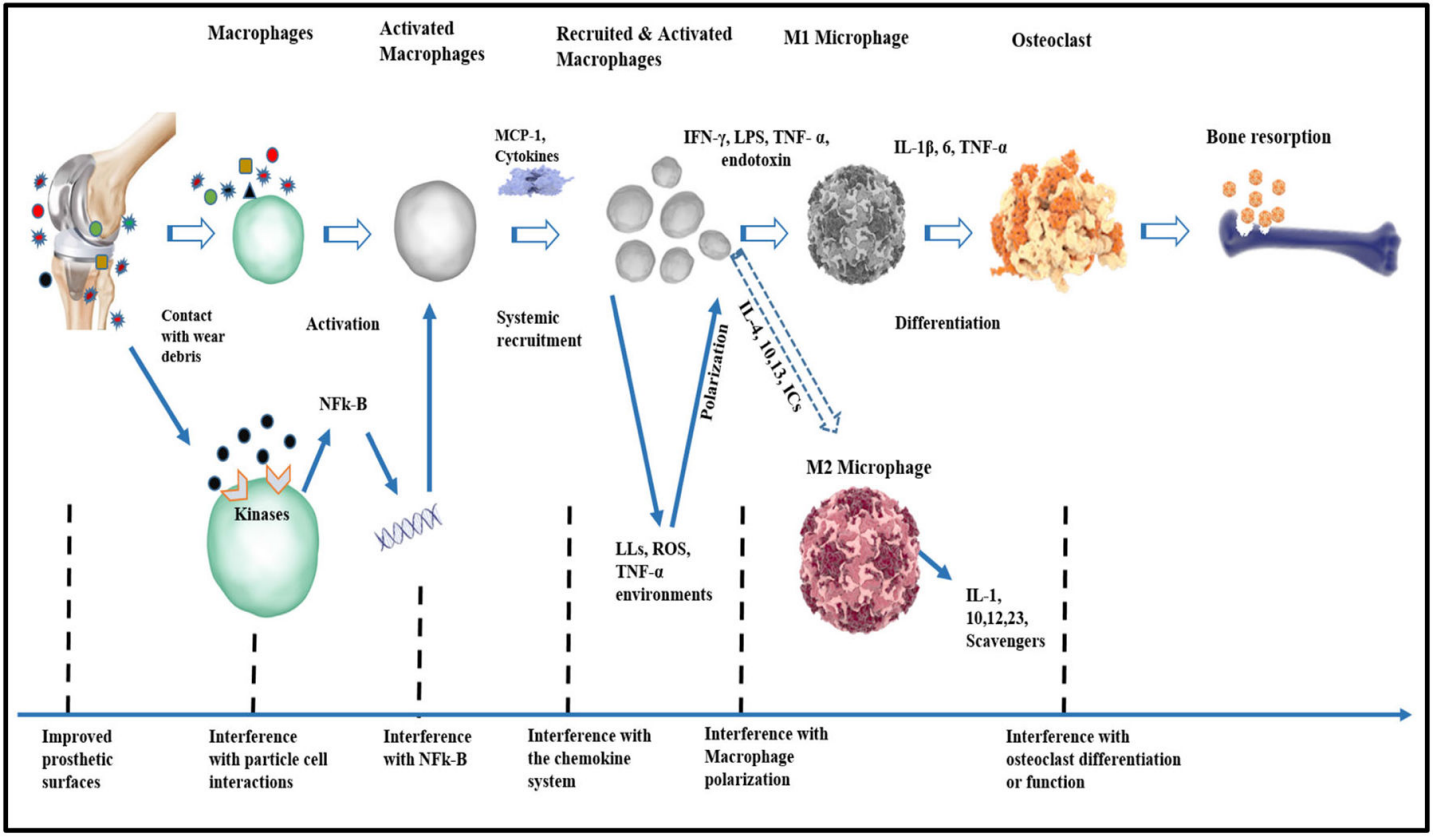
Biological strategies for the treatment of wear particle-induced metallosis and osteolysis. The schematic diagram outlined some possible biological approaches to preventing periprosthetic osteolysis owing to wear particles from implants. Macrophages (M2) as an attractive target for a wide variety of therapeutic interventions, as they are serve as key regulators of inflammation, immunity, tissue regeneration, and modulation of their activation states. The transition from a state of inflammation (M1) to tissue regeneration (M2) is presumed to depend on local dynamic shifts in the macrophage phenotype from the inflammatory M1 to M2 phenotypes. This functional plasticity/polarization represents a continuous polarization state rather than strict dichotomy

A proposed biological strategy that could mitigate metallosis is shown in Fig. 6. This approach perceived metallosis and osteolysis due to wear particles as a local biological phenomenon that could be modulated through local intervention. As depicted earlier in the “NF-kB Activation Leading to Inflammatory Osteolysis” section, the transition from a state of inflammation to tissue healing and restoration of function is presumed to depend on local dynamic shift in the macrophage phenotype from the inflammatory M1 to M2 phenotypes. This functional plasticity, now believed to be a continuous polarization state rather than strict dichotomy, makes macrophages an attractive target for a wide variety of therapeutic interventions, as they can serve as key regulators of inflammation, immunity, tissue regeneration, and modulation of their activation states [45, 48]. Macrophage contacts with wear debris create receptor kinases interference which stimulates NFK-b actions, and transformation of native macrophages to activated macrophage. These lead to recruitment of M1 cells and attraction of systemic pro-inflammatory agents, characterized by MCP-1, IFN-γ, LPS, TNF-α, endotoxin, etc. With further inflammatory actions (IL-1β, IL-6, and TNF-α), activated microphage could be different to osteoclast, causing bone resorption and implant failure [14, 47, 48, 50]. However, if the interference with the progressive migration of macrophages to the implantation site by inhibition of the chemokine-receptor axis would be initiated, thereby altering the functional activities of local macrophages by the conversion of pro-inflammatory M1 macrophages to anti-inflammatory pro-healing M2 phenotypes, then the modulation of the production and release of other harmful pro-inflammatory chemokines and cytokines would be averted through the inhibition of key transcription factor NF-kB (Fig. 6) [45].

Chelation therapy as a prophylactic intervention to prevent inflammatory responses has also been under intense investigation, in which most of the circulating unwanted metal ions are targeted [54, 55]. N-acetyl-cysteine and other chelating agents are given orally, intramuscularly, or intravenously; an administered chelating agent binds targeted metal ions and removes them from the body. However, excessive amounts of localized metal ions and particles produced in the joints spaces still proves difficult to chelate, limiting the success of this therapy on arthroplasty [55, 56].

Our group has developed and patented a polysaccharide moiety made up of chelating agents “chelator-functionalized glycosaminoglycans (dimercaprol-functionalized glycosaminoglycans).” The chelating agent is covalently bonded to the polysaccharide, which can include a hyaluronic acid polymer or a sulfated glycosaminoglycan moiety. The compound, which has demonstrated to be cytocompatible and possessing synovial fluid mimicking polymeric systems, can chelate cobalt, nickel, and chromium, making it a promising option to prevent or delay metallic debris-mediated implant failure and treat device-originated metallosis [57]. With these efforts in place, the future challenge is to devise joint replacement implants that will be integrated and function for the lifetime of the patient without any adverse biological and biotribocorrosion reactions.

## Figures and Tables

**Table 1:** List of metals used for implants and associated pathophysiology at toxic concentration

Metal	Major physiological roles	Deficiency manifestations	Manifestations in toxicity/excess	Other commercial uses
Essential metals				
Cobalt (Co)	Metabolism of purines/pyrimidines, amino acids, fatty acids, folate	Anemia Neuropathy Neuro-cognition changes	ACD, cardiomyopathy, polycythemia Altered thyroid function	Cobalamins (Vit B_12_), oxidation catalysts, pigment and coloration, radioisotopes, radioactive tracer, electroplating
Copper (Cu)	Collagen cross-linking Bone formation Iron metabolism Hemostasis/thrombosis Neurotransmitter synthesis Free radical control	Iron-refractory anemia Neutropenia/infection Osteoporosis Neurological dysfunction	GI symptoms Hemolysis Cardiac failure Renal failure Hepatic dysfunction Alzheimer’s	Medicine, bacteriostatic agents, fungicides, antibiofouling, electronics devices, wire, and cable
Iron (Fe)	Oxygen transport Oxygen storage DNA synthesis/repair RNA transcription Synthesis of collagen, neurotransmitters Energy metabolism Immune function	Microcytic anemia Diminished thyroid function Impaired neutrophil function Impaired cognition	Free radical generation Acute GI symptoms Hemochromatosis; cardiomyopathy; cirrhosis; diabetes; arthritis	Medicine, iron supplements, ferrioxalate, sewage treatment, catalyst, water purification, clothe dying, Prussian blue
Manganese (Mn)	Metabolism of carbohydrates, lipids Neurotransmitter synthesis Bone/cartilage formation Urea metabolism Control of free radicals	Dermatitis Weight loss Growth retardation Abnormal bone/cartilage Dyslipidemia Glucose intolerance	Headache Psychiatric symptoms GI symptoms Parkinson’s-like signs/symptoms	Catalysts, oxidizers, detoxification agents, essential to iron and steel alloy, aluminum alloy, pigments
Molybdenum (Mo)	Metabolism of amino acids Metabolism of purine/nucleotides, UA Metabolism of drugs/pro-drugs Metabolism of neurotransmitters	Urinary tract stones Acute renal failure, Myositis Mental changes/coma	Elevated uric acid/gout Secondary copper deficiency Reduced testosterone	High strength alloys, light bulbs filaments, medical imaging, mammography
Zinc (Zn)	Protein and carbohydrate metabolism Immune function Wound healing DNA synthesis and repair Control of free radicals Stabilization of protein structure Intracellular signaling	Skin/mucosa changes Decreased immune function Delayed wound healing Neurological dysfunction Bleeding abnormalities Osteoporosis Delayed growth	GI symptoms (acute) Copper deficiency Myeloneuropathy	Medicine; topical applications (diaper rash), anti-corrosion, use for alloy, galvanization, catalyst in rubber manufacture, toothpaste, and mouthwash
Chromium (Cr)	Glucose metabolism/-tolerance Lipid metabolism	Impaired glucose tolerance Abnormal lipids profiles Peripheral neuropathy	Cr3+: potential liver issues; potential kidney issues CR6+: respiratory symptoms; dermatitis/ulcerations; GI symptoms; Lung cancer	Dietary supplement, metal alloys; chemical refractory; pigmentation; magnetic compound; magnetic tape; metal polish
Vanadium (V)	Phosphate metabolism Insulin enhancement Lipid metabolism		GI symptoms Headache Weakness Tremor	Used for passivation of free metal against corrosion; Catalyst; Oxidizer; Redox battery
Nonessential metals				
Nickel (Ni)			Delayed hypersensitivity Acute: GI symptoms, headache, vertigo, vision changes Chronic: altered iron metabolism; cardiovascular, respiratory or kidney disease; alteration in hemostasis of calcium, magnesium, manganese, zinc	Alloys; electroplating; magnets; rechargeable batteries; used as mesh in gas diffusion electrode
Titanium (Ti)			Suppression of osteogenic differentiation Yellow nail syndrome	Has been used in sunscreens, anti-tumor preparations; pigments, addictive; coating
Aluminum (Al)			Osteomalacia Hepatic dysfunction Anemia Dialysis encephalopathy (dementia, myoclonus) Association with Alzheimer’s	Frequently used in antacids, toothpaste, antiperspirants, astringents; dental cement; water purification; catalyst for polymer; vaccines as immune adjuvants
Silver (Ag)			Local argyria (blue-gray skin or organ discoloration)	Antimicrobial; medical instruments; photographic; X-ray films; disinfectants; catheter
Palladium (Pd)			Lip edema Itching Respiratory symptoms	Dentistry (dental amalgam); hydrogen purification; catalyst; surgical instruments
Platinum (Pt)			Certain Pt-containing compounds may cause respiratory symptoms including kidney toxicity, hearing loss, bone marrow damage	Catalysts; laboratory equipment; dentistry equipment; chemotherapy; decomposition of hydrogen peroxide in water
Tin (Sn)			Acute: GI symptoms, headache Altered metabolism of zinc, iron, copper Cholesterol metabolism	Alloys; electroplating; optoelectronic application; food packaging (tin cans); Li-ion batteries; dental care products; treatment of gingivitis
Tungsten (W)			Certain compounds may antagonize molybdenum and copper	Alloys; catalysts; X-ray tubes; incandescent light bulbs; radiation shielding

Abbreviations: *ACD* allergic contact dermatitis; *GI* gastrointestinal; *UA* uric acid (refs: 3, 21-29
